# One Website to Gather them All: Usability Testing of the New German SKin Cancer INFOrmation (SKINFO) Website—A Mixed-methods Approach

**DOI:** 10.1007/s13187-022-02258-5

**Published:** 2022-12-30

**Authors:** Theresa Steeb, Julia Brütting, Lydia Reinhardt, Julia Hoffmann, Nina Weiler, Markus V. Heppt, Michael Erdmann, Astrid Doppler, Christiane Weber, Dirk Schadendorf, Friedegund Meier, Carola Berking, on behalf of the German Skin Cancer Council

**Affiliations:** 1 Department of Dermatology, University Hospital Erlangen, Friedrich-Alexander University Erlangen-Nürnberg, Ulmenweg 18, 91054 Erlangen, Germany; 2 Comprehensive Cancer Center Erlangen – European Metropolitan Region of Nürnberg, 91054 Erlangen, Germany; 3 Skin Cancer Center at the University Cancer Centre Dresden and National Center for Tumor Diseases, Dresden, Germany; 4 Department of Dermatology, University Hospital Carl Gustav Carus, TU Dresden, Dresden, Germany; 5 Center for Evidence-Based Healthcare, University Hospital Carl Gustav Carus, Technical University Dresden, Dresden, Germany; 6 Eurice - European Research and Project Office GmbH, Ingbert, Germany; 7 Melanom Info Deutschland e.V. (MID), Essen, Germany; 8 MEDEA GmbH - Medical Education Academy, Hamburg, Germany; 9 Department of Dermatology, University Hospital Essen, University Duisburg-Essen, Essen, Germany

**Keywords:** Skin cancer, Usability testing, Qualitative research, Think-aloud, Melanoma, Internet

## Abstract

Skin cancer patients increasingly search the internet to acquire disease-related information. However, information on the internet may be misleading. Recently, SKINFO has been launched, a website exclusively created for German-speaking skin cancer patients providing information as well as additional resources of verified quality. Here, we describe the results of the first usability test of SKINFO using a mixed-methods approach. Ten adult patients with skin cancer were recruited for usability testing in the skin cancer units of the University Hospitals of Erlangen and Dresden, Germany. Testing consisted of three different scenarios where patients were asked to find specific information on the SKINFO website guided by the think-aloud method. Descriptive analysis and content analyses were performed. All patients would recommend SKINFO and appreciated its content, design, and structure. Think-aloud analysis identified the topics layout, navigation, and content and structure which would benefit from refinement. Major criticism included the navigation through the website, and the desire for more specific information addressing patients’ relatives and the latest, up-to-date information. Overall, usability testing showed that the unique web-based information platform has the potential to support patients coping with skin cancer and thus strengthen informed decision-making.

**Supplementary Information:**

The online version contains supplementary material available at 10.1007/s13187-022-02258-5.

## Introduction

Skin cancer patients have a high unmet need for disease-related information, education, and additional information about various disease-related topics [1]. Personal and particularly the physician-patient conversation remain the most important source of information [2]. Internet-based services, like websites, apps, and videos, have now replaced the primary use of print media like patient booklets [2]. However, the abundance of available information may overwhelm patients. Besides, the assessment of the respective accuracy and timeliness of the content due to lacking health literacy represents a major problem. Currently available German websites, booklets, and YouTube™ videos addressing patients with skin cancer have been found to be of predominantly mediocre quality and poor reliability [3–5]. Moreover, access to information is often restricted, resulting in an inability to satisfy the individual information needs [1, 2]. However, being informed about one’s disease is crucial, as it is the basis for shared decision-making and is considered not just a useful complement but a central necessity for comprehensive physician-patient communication and treatment adherence [6, 7].

Consequently, many patients wish for a freely accessible website that provides reliable and understandable information. Therefore, an interdisciplinary team developed the freely accessible website entitled “SKINFO” (Skin Cancer Information platform, available at: www.infoportal-hautkrebs.de), which was launched in February 2021. The website aims at German-speaking skin cancer patients, their relatives, and the general population. Information of verified quality is provided understandably on the diagnosis, treatment, and surveillance of different skin cancer entities as well as prevention and other patient-relevant topics such as psychosocial support, reimbursement, lifestyle, and fertility. Moreover, the website offers information on events, news from congresses, and the latest research outcomes.

Here, we describe the results of the websites’ usability test using a mixed-methods approach to test the utility and acceptability and to incorporate views and feedback of patients affected by skin cancer [8].

## Material and Methods

### Study Design

A usability test with semi-structured interviews and self-administered questionnaires was conducted between February 2021 and June 2021 at the skin cancer units of the University Hospitals of Erlangen and Dresden, Germany. Overall, ten patients (*n* = 5 at each center) were recruited by treating physicians (FM, ME). Previous research has shown that five users are sufficient to identify most usability problems [9]. The reporting of this research project was guided by the Standards for Reporting Qualitative Research [10].

### Eligibility Criteria

Patients were eligible for usability testing if they (1) were affected by any type of skin cancer, irrespective of the stage, (2) had been diagnosed within the last 5 years, (3) were at least 18 years of age, (4) had a very good understanding of the German language (native speaker level), and lastly (5) were familiar with using the internet.

### Usability Test

Patients met individually with the interviewer (TS, LR) to complete the test using the SKINFO website. They were asked to find specific information on the website: The first scenario included the search for a PDF document about adjuvant therapy options for melanoma. The second scenario covered possible cash benefits in case of skin cancer as an occupational disease, and the third scenario contained information on travel cost reimbursement (Supplementary Table 1). These scenarios were chosen as patients are often interested in these kinds of skin cancer–related information. Patients were encouraged to speak out loud about their thoughts and experiences while browsing the website when completing the tasks. This method represents a well-established, effective technique in the qualitative evaluation of websites [8, 11]. After the completion of the different tasks, patients judged the difficulty to solve each of the individual tasks on a scale from 1 (very difficult) to 7 (very easy).

Additionally, the patients filled in the German version of the User Experience Questionnaire (UEQ) [12, 13]. The questionnaire consists of randomized pairs of opposites to measure the users’ experiences interacting with a prototype. It covers six domains with 26 items that assess the following characteristics on a scale of 1 to 7: (1) *Attractiveness:* overall impression of the website, whether the patients like or dislike the website. Items included in this scale are annoying/enjoyable, good/bad, unlikeable/pleasing, unpleasant/pleasant, attractive/unattractive, friendly/unfriendly. (2) *Perspicuity*: Ask the patients whether they understand how to use the website and are familiar with it. Items included in this scale are not understandable/understandable, easy to learn/difficult to learn, complicated/easy, clear/confusing. (3) *Efficiency*: Identifying whether the patients are able to solve their tasks fast and efficiently without unnecessary effort. Items included in this scale are fast/slow, inefficient/efficient, impractical/practical, organized/cluttered. (4) *Dependability*: Understanding whether the patients feel in control of the interaction. Items included in this scale are unpredictable/predictable, obstructive/supportive, secure/not secure, meets expectations/does not meet expectations. (5) *Stimulation*: Identify whether the website is interesting and exciting to the patients. Items included in this scale are valuable/inferior, boring/exciting, not interesting/interesting, motivating/demotivating. (6) *Novelty*: Identify whether the website is innovative and creative enough for the patients and the ability of the website to gain attention from the patients. Items included in this scale are creative/dull, inventive/conventional, usual/leading-edge, conservative/innovative [12, 13].

Besides, the participants provided socio-demographic data via a self-administered questionnaire. Additionally, a questionnaire covering aspects of satisfaction with the website, including navigation, content, and impression was distributed (rating on school grades between 1 (best) and 6 (worst)). Patients were asked whether they would recommend the website to others or would use the website at home. Finally, patients were also invited in a free-text field to comment on missing aspects of the website or aspects that need improvement, as well as things they liked.

### Consent

All patients were identified by pseudonyms to ensure anonymity. Prior to the interviews, patients were informed about the study’s purpose, procedure, and data protection. All patients provided written consent. They received an incentive of €80 as reimbursement for their time.

### Data Analysis

All think-aloud interviews were audiotaped and transcribed verbatim by TS and LR. Following the transcription of the interviews, a thematic analysis was performed by TS. The quotes were categorized according to an inductive approach [14, 15]. All interviews were conducted in German. Selected quotes were translated by a native speaker. Socio-demographic data, as well as the data from closed-ended questions, were summarized descriptively. UEQ data were evaluated using the corresponding data analysis tool provided in Microsoft Excel 2007 [13, 16]. Data for the values per item were transformed to values ranging from + 3 to − 3 to represent the most positive and most negative value [13]. Results were summarized using descriptive statistics, including mean and standard deviations. For the purpose of comparison, previously established and published benchmark data was used utilizing the same Excel spreadsheet as for the other calculations [12, 13]. The comparison of the results for the SKINFO website with the data in the benchmark as external control allows preliminary conclusions about the relative quality of the website in general compared to other products.

## Results

### Baseline Characteristics

Six women and four men with a median age of 54.5 years (range 27–67) participated in the usability test (Table 1). All of them had been diagnosed with melanoma; half of them were in advanced stages. One patient had been additionally affected by basal cell carcinoma and actinic keratosis. The duration of the interviews ranged from 11:33 to 41:53 min. The majority of patients answered to search less than once a month for disease-related information, while one patient stated to look up information 2–3 times per week. Most patients indicated to have searched for information on the internet after they had received their diagnosis and looked for information related to skin cancer, its causes and prevention (*n* = 8). The majority uses search engines with specific keywords (*n* = 5).

**Table 1:** Overview of the characteristics of the 10 patients

	Skin cancer unit	Length of the interview (min/s)	Sex	Age	Type of skin cancer	Year of diagnosis	Information seeking behavior	Residence
1	Erlangen	16:53	Female	45	Primary cutaneous melanoma	2016	< once/month	Urban
2	Erlangen	19:38	Female	57	Primary cutaneous melanoma, basal cell carcinoma, actinic keratoses	2019	< once/month	Outskirts
3	Erlangen	23:18	Male	58	Primary cutaneous melanoma	2020	< once/month	Outskirts
4	Erlangen	12:31	Female	27	Primary cutaneous melanoma	2017	< once/month	Urban
5	Erlangen	41:53	Male	52	Advanced cutaneous melanoma	2019	< once/month	Rural
6	Dresden	11:33	Female	37	Primary cutaneous melanoma	2020	< once/month	Urban
7	Dresden	22:12	Male	67	Advanced cutaneous melanoma	2017	< once/month	Rural
8	Dresden	25:24	Male	65	Advanced cutaneous melanoma	2020	< once/month	Rural
9	Dresden	17:23	Female	42	Advanced cutaneous melanoma	2018	2–3 times/week	Rural
10	Dresden	19:09	Female	64	Advanced cutaneous melanoma	2016	< once/month	Rural

### General Impression of the Website

**Fig. 1:**
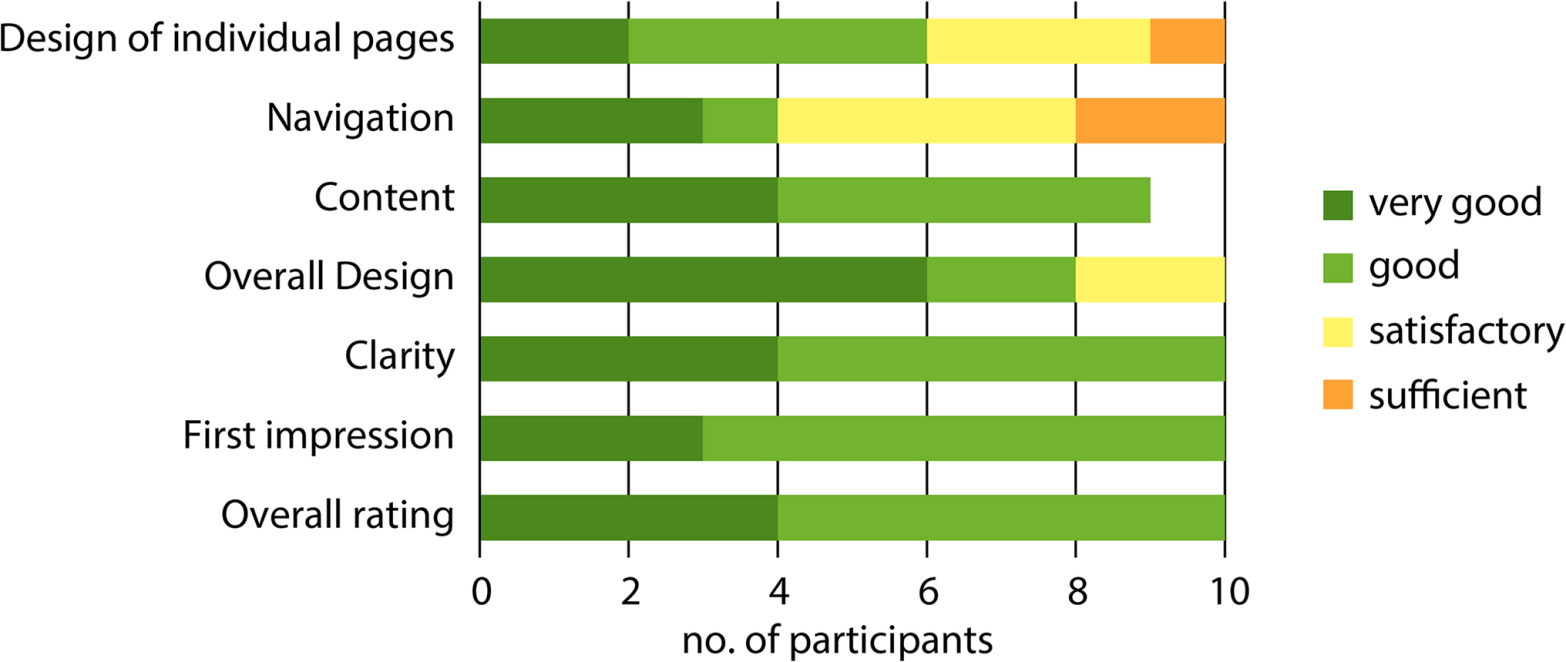
Bar chart showing patients’ school grading (1 (very good)–6 (deficient)) of the properties of the SKINFO website

Overall, all patients appreciated SKINFO. The website has evoked interest in all patients (10/10). All patients indicated that they would use the website personally and recommend SKINFO. The majority of patients (6/10) rated the website generally to be good, and the remaining patients even very good (*n* = 4) (Fig. 1). The first impression was also either very good (3/10) or good (7/10). Nearly all patients judged the website trustworthy, except for one patient who criticized that no general contact details were available at the time of the usability testing. Besides this, all patients considered the website to be clear (very good: 4/10, good: 6/10) and the content to be good (very good: 4/9, good: 5/9, *n* = 1 missing). Two patients rated the overall design to be sufficient, two rated it to be good and another six to be very good. However, the design of the individual pages of SKINFO was judged as sufficient by one patient, satisfactory by three patients, good by four patients, and very good by two patients. Navigation was rated lowest among all items (very good: 3/10, good: 1/0, satisfactory: 4/10, sufficient: 2/10).

### Usability Tasks

**Fig. 2:**
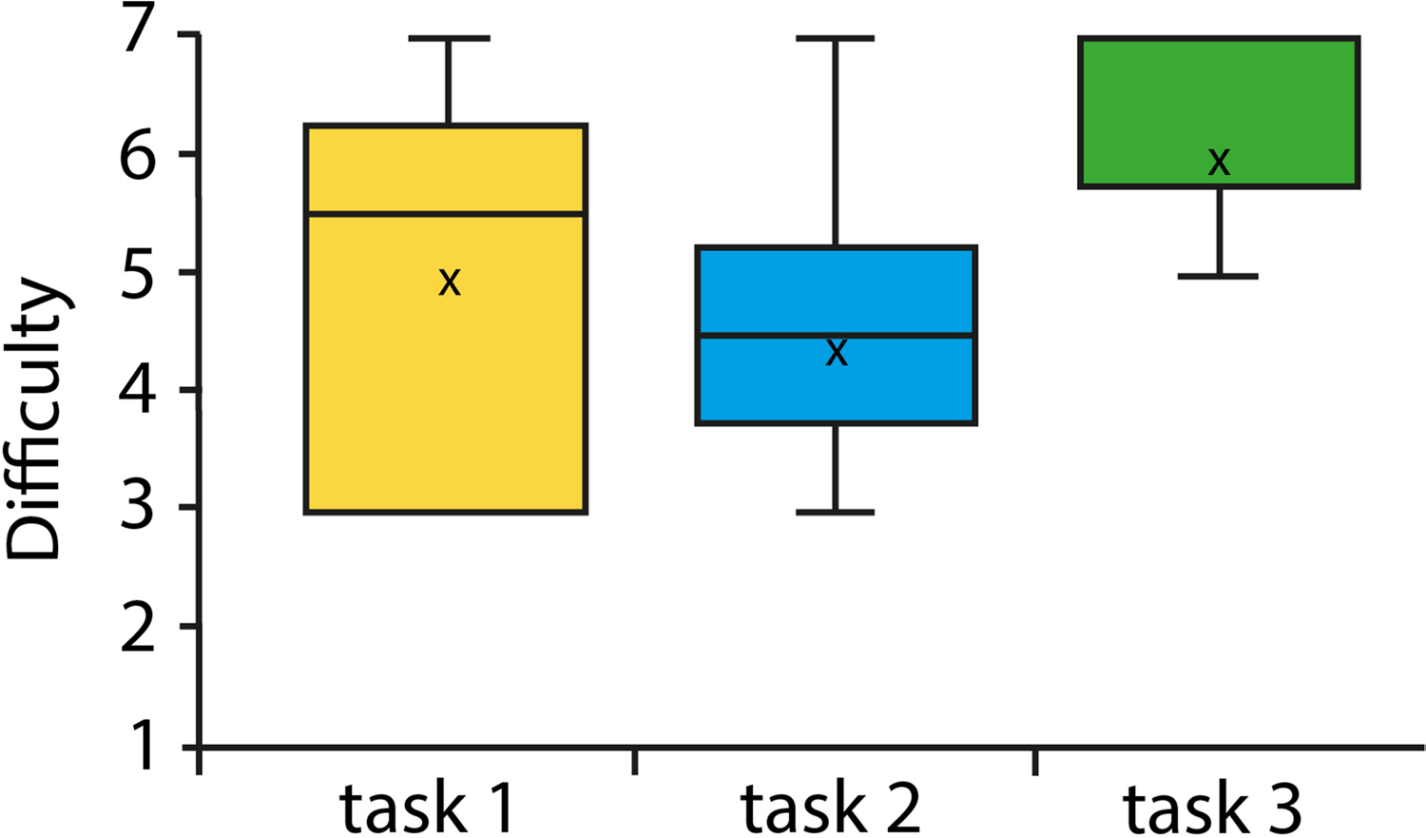
Difficulty of the three different usability test scenarios ranging from 1 (very difficult) to 7 (very easy)

Overall, all patients perceived the final task on the travel reimbursement to be the easiest with a mean score of 6 (± 1.89) while the second task on cash benefits was rated the most difficult (mean score: 4.4 ± 1.65) (Fig. 2). The task related to the identification of the melanoma brochure was on average rated to be easy with a mean score of 5 (± 1.63), however with the most variability in the rating.

The patients’ comments from all think-aloud testing scenarios were grouped into the topics (1) layout, (2) navigation, and (3) content and structure [14]. The major aspects from these themes and a selection of quotes are summarized in Supplementary Table 2.

### Layout and Navigation

The main problems identified concerning the websites’ layout were the usability of the drop-down menu and the contrast of the color scheme and font. In particular, the font was identified to be sometimes too small and hard to read due to the chosen color as well as hard to distinguish from the referenced literature due to the size.

The most prominent problem relating to the websites’ navigation was the horizontal arrangement of the subcategories. Nearly all patients overlooked it and criticized that too many intermediate clicks were necessary to navigate the website.

So you had to click for a long time until you got somewhere […] there was not the possibility to go directly to a tab (Pat. 4)

### Content and Structure

During the think-aloud sessions, the patients identified problems regarding the website’s content and structure, for example, the assignment of the contents to the given categories.

One patient remarked that no contact address was available, which limits the websites’ professionalism and trustworthiness. Another patient wished more detailed information on melanoma stages and their meaning. Another aspect identified was that no information for patients’ relatives and their specific needs was provided on the website, although SKINFO claims to address skin cancer patients’ relatives and all interested parties.

The heading [implies that this webpage] is also for affected persons, relatives, interested parties […] But I do not find that [specific information] in there (...). Infoportal for affected people, that's what I found, but not for the relatives and interested people (...) because, well, from my personal experience, when you come home and tell your family you have skin cancer, then other family members are worried. They are not affected, but they would like to know how they can help the affected [person] (...) and I don't find that [information] anywhere in here. (Pat. 5)

Besides this, one patient mentioned missing current knowledge and the latest news regarding skin cancer prepared in an understandable language for laypersons.

[…] to be able to participate in this knowledge. Let’s say, a little bit like Drosten [=Christian Drosten, German virologist] did with Corona. I think his greatest merit is that he translates the state of science (...) for the general public in such a way that you can understand it with a little bit of goodwill. But he keeps up with the current developments. He also sometimes says, we don’t know yet, we’re doing research, a new study has just been published, but I evaluate it this way and that way. (Pat. 3)

### Further Strengths, Weaknesses, and Wishes

In a free-text field, patients could highlight aspects they especially liked as well as potential limitations of the website. Most patients appreciated the versatile content and detailed explanations, the arrangement of the topics, the colorful design, and the up-to-dateness, particularly the news part. One patient also appreciated the clarity of the website, and that information was easy to find. In contrast, potential weaknesses mainly referred to the navigation of the website through the drop-down menu and the horizontal navigation, respectively, as expressed during the think-aloud scenarios. Besides, one patient stated to miss a general overview from the beginning, which information can be found under which tab. Another one pointed out that some things are hard to find.

### UEQ

The highest mean values ± standard deviations were awarded for the sub-scales stimulation (2.2 ± 0.695), dependability (2.092 ± 0.654), and attractiveness (2.003 ± 0.783), while novelty (1.125 ± 1.029) and perspicuity (1.625 ± 1.276) were scored lowest. Efficiency yielded an intermediate mean score (1.933 ± 0.968). A detailed summary of the individual pairs of opposites comprising the subscales is presented in Supplementary Table 3. In the benchmark comparison, the website’s attractiveness, efficiency, dependability, and stimulation were in the range of the 10% best results compared to other websites and thus scored as excellent (Supplementary Fig. 1).

## Discussion

SKINFO was launched in February 2021 and addresses German-speaking skin cancer patients, their relatives, and interested parties. The purpose of the website is to provide high-quality and reviewed information — independent from commercial interests — on common and less common skin cancer entities as well as information on further patient-related topics. Usability testing represents an important step in the creation and optimization of patient information as it enables the detection of substantial deficits [14, 17–20]. This first usability testing represents an important milestone by incorporating feedback from the target users, i.e., patients affected by skin cancer.

Overall, the feedback was positive, and all patients would recommend SKINFO and appreciated its content, design, and structure. Interestingly, the last task was rated as the easiest by all patients. This may be explained by the fact that the patients first had to get familiar with the website and examine its topics and the navigation.

Think-aloud analysis revealed that the identified topics layout, navigation, and content and usefulness need modification. Regarding the content, one of the main limitations was that the webpage claims to aim at skin cancer patients and their relatives. However, the study participants had the impression that this was not the case (“The heading [implies that this webpage] is also for affected persons, relatives, interested parties […] But I don’t find that [specific information] in there”, Pat. 5). Importantly, the relatives of patients also have information and support needs, which are not met yet. Considering their views and information need is especially important, as being informed about the disease enables them to support the patients’ preferences for care and deal with practical demands and difficulties in everyday life [21]. Especially information deficits regarding financial support have been expressed [22]. A recent study showed that the internet is the second most preferred information source after the oncologist among cancer patient relatives [23]. Thus, it is crucial to optimize the web page and include a distinct category addressing the specific needs of relatives [21, 22, 24]. Consequently, relatives should also be part of the next usability testing of the website.

Furthermore, SKINFO yielded good results in the evaluation with the UEQ and was in the range of the 10% best results compared to other websites for the items attractiveness, efficiency, dependability, and stimulation. However, these benchmark comparison results have to be interpreted cautiously as they comprise a sample of various websites with different content focus [12].

In previous studies, available online information for German melanoma patients including web pages or videos was evaluated to be of mediocre quality, good usability, and understandability but low reliability and even very low readability [3, 5], while a medium quality, a high application of understandability elements, but low readability was found when booklets were rated [4]. Most deductions could be explained by incomplete reporting on treatments and insufficient meta-information [3, 5]. One important asset of SKINFO is the preparation of understandable and reliable skin cancer–related information for laypersons, the continuous dissemination of current knowledge and news as well as the link to already existing videos, web pages, and brochures that have been validated regarding their quality. One patient substantially highlighted the urgent need for the latest information prepared in an understandable manner. Thus, the preparation and dissemination of information on recent scientific progress on skin cancer, for example from conferences and scientific publications, in an understandable but still reliable way is one of the ultimate goals of SKINFO and should be continually pursuit.

Of note, the interdisciplinary project team will discuss the identified problems and possible solutions, such as simplifying the navigation and including the visibility of the menu button. The adjustments will be discussed as part of a consensus meeting. Nevertheless, another usability test should be performed in the future to check the implementation of the criticized aspects proposed in this current usability test. Furthermore, continuous testing and obtaining feedback from patients, their relatives, physicians, and researchers should be realized in order to guarantee steady improvement of the website. Five patients were recruited at the University Hospital in Dresden and another five at the University Hospital of Erlangen. According to previous research, five users are sufficient to identify most usability problems in a similar context [9]. Thus, the recruitment in our study exceeded the desired sample size and includes the views of patients located in two geographically different parts of Germany (Bavaria and Saxony). Besides, the sample was quite heterogeneous, representing the variety of patients affected by skin cancer and the target group of the website, although melanoma patients were overrepresented. However, the next usability test should focus on recruiting patients affected by non-melanoma skin cancer as well.

## Supplementary Information

ESM 1 (JPG 464 kb)

ESM 2 (DOCX 13 kb)

ESM 3 (DOCX 22 kb)

ESM 4 (DOCX 19 kb)

## Data Availability

Data available on request from the authors: the data that support the findings of this study are available from the corresponding author (TS), upon reasonable request.

## References

[1] Brütting J, Bergmann M, Garzarolli M (2019). Unmet information needs of patients with melanoma in Germany. Melanoma Res.

[2] Brütting J, Bergmann M, Garzarolli M (2018). Information-seeking and use of information resources among melanoma patients of German skin cancer centers. J Dtsch Dermatol Ges.

[3] Brütting J, Steeb T, Reinhardt L (2018). Exploring the most visible German websites on melanoma immunotherapy: a web-based analysis. JMIR Cancer.

[4] Brütting J, Reinhardt L, Bergmann M (2019). Quality, readability, and understandability of german booklets addressing melanoma patients. J Cancer Educ.

[5] Steeb T, Reinhardt L, Gorgmayr C (2020). German YouTube videos as a source of information on cutaneous melanoma: a critical appraisal. J Eur Acad Dermatol Venereol.

[6] Elwyn G, Frosch D, Thomson R (2012). Shared decision making: a model for clinical practice. J Gen Intern Med.

[7] Frosch DL, Kaplan RM (1999). Shared decision making in clinical medicine: past research and future directions. Am J Prev Med.

[8] Maramba I, Chatterjee A, Newman C (2019). Methods of usability testing in the development of eHealth applications: a scoping review. Int J Med Inform.

[9] Turner C, Lewis J, Nielsen J, Karwowski W (2006). Determining usability test sample size. International Encyclopedia of Ergonomics and Human Factors.

[10] O'Brien BC, Harris IB, Beckman TJ (2014). Standards for reporting qualitative research: a synthesis of recommendations. Acad Med.

[11] Jaspers MW (2009). A comparison of usability methods for testing interactive health technologies: methodological aspects and empirical evidence. Int J Med Inform.

[12] Schrepp M, Hinderks A, Thomaschewski J (2014). Construction of a benchmark for the User Experience Questionnaire (UEQ). Int J of Interact Multimedia and Arti Intel.

[13] Schrepp M, Hinderks A, Thomaschewski J (2014) Applying the User Experience Questionnaire (UEQ) in different evaluation scenarios. In: Marcus A (ed) Design, User Experience, and Usability. Theories, Methods, and Tools for Designing the User Experience. Lecture Notes in Computer Science, vol 8517. Springer International Publishing, pp 383–392

[14] Dauber-Decker KL, Basile M, King D (2021). Developing a decision aid to facilitate informed decision making about invasive mechanical ventilation and lung transplantation among adults with cystic fibrosis: usability testing. JMIR Hum Factors.

[15] Mayring P (2010) Qualitative inhaltsanalyse. In: Mey G, Mruck K (eds) Handbuch Qualitative Forschung in der Psychologie. VS Verlag für Sozialwissenschaften

[16] Rauschenberger M, Cota MP, Thomaschewski J (2013). Efficient measurement of the user experience of interactive products. Int J Interact Multimedia Arti Intel.

[17] Babatunde FO, MacDermid J, Grewal R (2020). Development and usability testing of a web-based and therapist-assisted coping skills program for managing psychosocial problems in individuals with hand and upper limb injuries: mixed methods study. JMIR Hum Factors.

[18] Hoffmann M, Taibinger M, Holl AK (2019). Online information for relatives of critically ill patients : pilot test of the usability of an ICU families website. Med Klin Intensivmed Notfmed.

[19] Hoffman AS, Bateman DR, Ganoe C (2020). Development and field testing of a long-term care decision aid website for older adults: engaging patients and caregivers in user-centered design. Gerontologist.

[20] Starling R, Nodulman JA, Kong AS (2015). Usability testing of an HPV information website for parents and adolescents. Online J Commun Media Technol.

[21] Foster C, Myall M, Scott I (2015). 'You can't say, "what about me?" I'm not the one with cancer': information and support needs of relatives. Psychooncology.

[22] Eriksson E, Arve S, Lauri S (2006). Informational and emotional support received by relatives before and after the cancer patient's death. Eur J Oncol Nurs.

[23] Yildirim S, Kazaz SN, Semiz HS (2019). An evaluation of the information sources of cancer patients' relatives. a prospective survey. J Cancer Educ.

[24] Pinkert C, Holtgrawe M, Remmers H (2013). Needs of relatives of breast cancer patients: the perspectives of families and nurses. Eur J Oncol Nurs.

