# Supplementary figures and images for: One Website to Gather them All: Usability Testing of the New German SKin Cancer INFOrmation (SKINFO) Website—A Mixed-methods Approach
Source: J Cancer Educ. 2022 Dec 30;38(4):1264–70. doi: 10.1007/s13187-022-02258-5 (PMC10366310; doi:10.1007/s13187-022-02258-5)

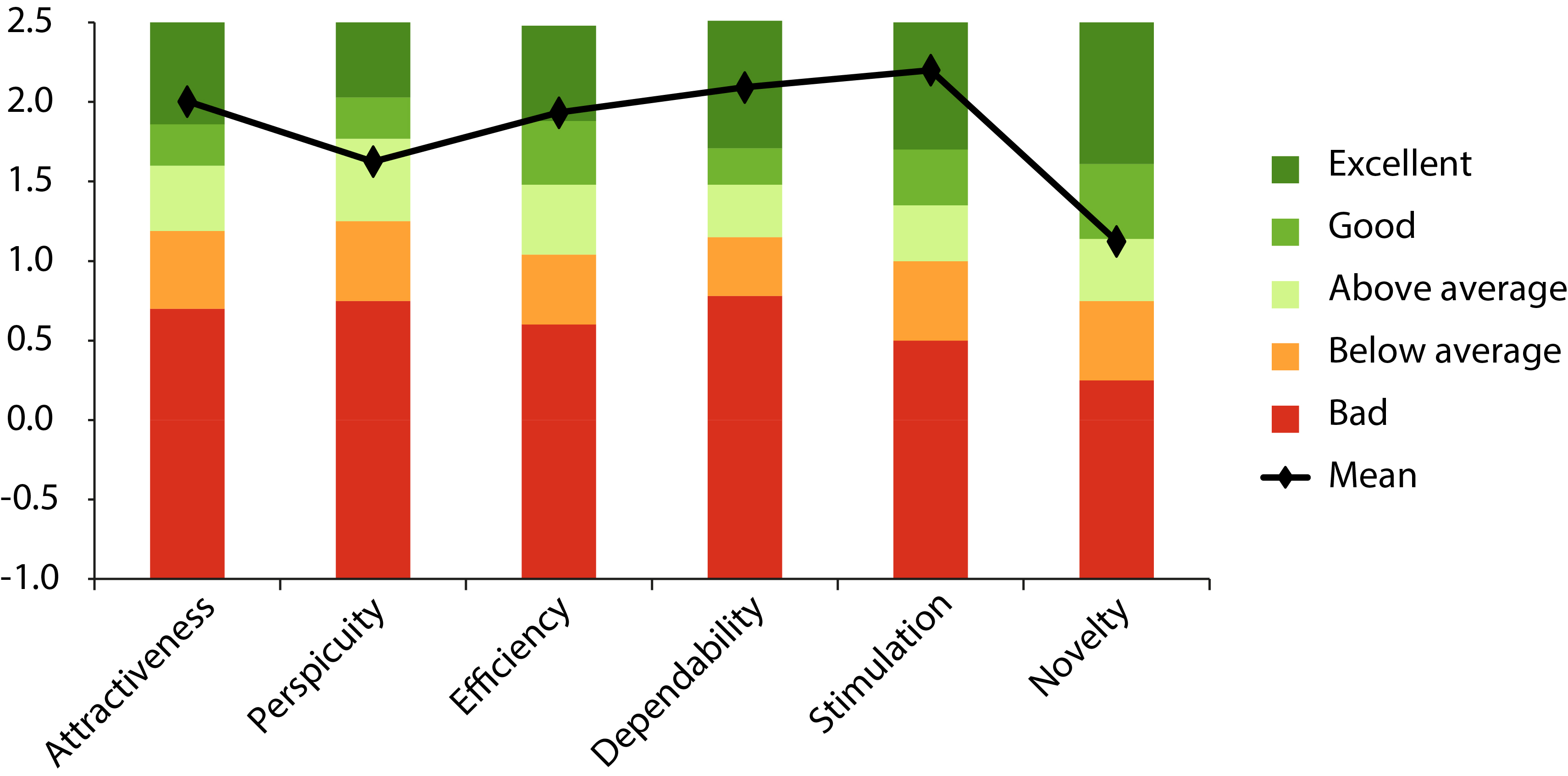

Supplement: Supplementary file 1 — (JPG 464 kb) [file 13187_2022_2258_MOESM1_ESM.jpg]
