# Supplementary material for: One Website to Gather them All: Usability Testing of the New German SKin Cancer INFOrmation (SKINFO) Website—A Mixed-methods Approach
Source: J Cancer Educ. 2022 Dec 30;38(4):1264–70. doi: 10.1007/s13187-022-02258-5 (PMC10366310; doi:10.1007/s13187-022-02258-5)
Supplement: Supplementary file 2 — (DOCX 13 kb) [file 13187_2022_2258_MOESM2_ESM.docx]

**Supplementary Table 1: Overview of the three usability task scenarios.**

| **Task 1** | “Imagine your doctor has recommended a brochure on adjuvant therapy for melanoma. I'll say something else about it in a minute. Unfortunately, he didn't have any more copies of it at hand. However, he recommended downloading it from the website. So find the brochure on adjuvant therapy for melanoma and proceed as you would outside of this test.” |
| --- | --- |
| **Task 2** | “Certain types of skin cancer may develop due to someone’s professional activity; therefore, they may be recognized as an occupational disease. Please search the website for the cash benefits to which you may be entitled in such a case. Proceed as you would outside of this test. The task is complete when you have found the possible cash benefits. So, please search for skin cancer as an occupational disease and what cash benefits you may be entitled to.” |
| **Task 3** | “You have heard that travel expenses that are related to your skin cancer therapy may be reimbursed under certain conditions. Look for information on travel reimbursements and proceed as you would outside of this test.” |
