# Supplementary material for: One Website to Gather them All: Usability Testing of the New German SKin Cancer INFOrmation (SKINFO) Website—A Mixed-methods Approach
Source: J Cancer Educ. 2022 Dec 30;38(4):1264–70. doi: 10.1007/s13187-022-02258-5 (PMC10366310; doi:10.1007/s13187-022-02258-5)
Supplement: Supplementary file 3 — (DOCX 22 kb) [file 13187_2022_2258_MOESM3_ESM.docx]

**Supplementary Table 2: Common themes and selected quotes from the three different scenarios (n = 10 patients)**

| **Themes** | **Sub-category and quotes** |
| --- | --- |
| **Layout** | **Identification of the drop-down menu and its appearance**   - *"Actually, this sign [=menu button] is familiar from cell phone apps. Now, on a normal computer [...] I didn't react to it at all."* (Patient #3) - *"You have to say that this [icon] is a [menu] button [...] Then it also has the same color as the text, the blue, but it has to stand out [to be recognizable]."* (Patient #5) - „*I really just came across it [=drop-down menu] accidentally. The menu button wasn't that obvious. It's rather good actually, but you can also overlook it.”* (Patient #6) |
|  | **Contrast of the color scheme and font as well as font size**   - „[…] *but these colors just do not fit together in my opinion*“ (Patient #5) - *"[The icon] “Current knowledge”. Yes, that's hard to read. Very hard to read compared to [the icons] “About us”, “Events”, “News”.“* (Patient #5) |
| **Navigation** | **Horizontal navigation was overlooked, too many clicks were necessary to navigate through the website.**   - *"(...) You don't get to the information via a vertical hierarchy like I go there and there and then there, but you have to get the idea that you move on horizontally in a certain chapter; however, you don't see the next but one and the next but one page after that in that horizontal [navigation mode]. Therefore, you don't know if this is the right way. You can try it, but you don't know if you'll get there [to the desired information]. Because it were 6, 7 or let's say 5 steps that I had to move horizontally until I luckily landed at the right information. Thus, it would be easier for me if I could see a list [=dropdown menu], so to speak (...) at [the icon] daily life with skin cancer” and then [the heading] “possible financial benefits” or whatsoever. Ah, then I don't need to go through 5 steps, but can go directly to the step that interests me."* (Patient #3) - „*So, here I found it a bit difficult that you always had to click through [the chapter] completely. To be honest, as a normal internet user, I just find that [information] somehow, […] I don't have an overview here, what's actually all here now, but that I always have to click [at the icon] on the right-hand side at the bottom of the page [to see what’s coming next].*” (Patient #4) - „*Well, you had to click for a long time until you got somewhere, you really had to look at everything, so there was no possibility to go directly to a tab where you can read about money.*“ (Patient #4) - „*Well, it was not so easy to find, so you have to really think where it [=the information] could be and everywhere, many intermediate clicks then you come [to the information], yes. (...) But now as a first impression, you have to click your way in quite a bit first.“* (Patient #9) |
|  | **You have to scroll down on the front page to see all icons.**   - „*I thought it [=the front page] does not respond, but you just need to scroll down*“ (Patient #9) |
| **Content and structure** | **Some of the content was assumed to be listed in other categories**   - *For me, it was not quite understandable, why this [topic] was listed in [the category] “current knowledge” ”* (Patient #1) |
|  | **Category “downloads” should be made visible on the front page of SKINFO as an extra category**   - „*Well, first of all, I look at the headings that are here at the portal page. So, from “skin cancer types” to “news”. And (...) if I had found a section called "downloads" or "information", I would have clicked on it. I can't find it there.“* (Patient 3) - „*Now, that was a random hit that I found the [subcategory] “downloads” in [the category] “knowledge”. If you are on normal [webpages], Iet’s say shopping pages or of any products, you will usually find a section "service, downloads" (…), where you can then access the available materials relatively quickly.”* (Patient 3) |
|  | **More detailed information on melanoma stages and their meaning**   - *„But what would have been interesting for me now, for example, is this [melanoma] staging. What does this mean, for example? I have already tried to find out and […] I'm not a fan of using that Google [search engine for that], because then you just get a bunch of stuff. Either you're a doctor and understand that or you just let it be. I have not really found a reliable [...] - let’s say – simple, straightforward answer for non-medics, that would be at least somehow credible. Therefore, I do not deal with the stuff in-depth, because if I want to know something, I ask my doctor. (...)“* (Patient #7) |
|  | **One part of the website only dedicated to the category of costs and reimbursement instead of having several subcategories addressing these different forms of financial aspects**   - *“Why are, for example, the travel costs now [listed] here (…) [but] not still below. Why do you have to go to the right [=next page] now? These are all cost categories, travel costs are on a new page”* (Patient #3) |
|  | **Information especially for patients’ relatives and their needs was provided on the website**   - "*The heading [implies that this webpage] is also for affected persons, relatives, interested parties […] But I don't find that [specific information] in there (...) when I have the heading. Infoportal for affected people, that's what I found, but not for the relatives and interested people (...) because, well, from my personal experience, when you come home and tell your family you have skin cancer, then other family members are worried. They are not affected, but they would like to know how they can help the affected [person] (...) and I don't find that [information] anywhere in here*. […]*. Does she [=patient’s relative] have to drive me, can I still [drive], if I have chemo [=chemotherapy] now, does she have to accompany me? Am I still fit to drive?"* (Patient #5) |
|  | **Current knowledge and latest news regarding skin cancer prepared in an understandable language for laypersons**   - *"So, at some point, as a user, you get to the point where you say, now I would like to have even more precise [information], now I would like to have, for example, primary source information. And I'm not a medical doctor, so I don't really have any idea, but I've noticed that with a bit of concentration, you can definitely read and understand such medical texts. And (...) I could also imagine that it would be helpful for many people if, for example, there were good studies or helpful studies that could bring more transparency into the process, if they were accessible in a prepared form via such a portal. (...) This portal but also (...) [the] German Cancer Research Center […] [webpage] is beautifully prepared in the sense of simplification for laypersons etc. and then you come to a certain level of information and then you can’t go further. Then comes “yeah, yeah” and “everything else discuss with your doctor”. That’s it. Well, I sometimes want to inform myself further, perhaps also as preparation for the doctor-patient discussion, that I know exactly what I can ask. Or in my case, it was like this, the doctor told me, well, here is the famous “poison sheet”, listing all side effects that can occur. Then I wanted to know which of these side effects can be monitored well by consistent observation to allow early interventions, if they [=side effects] should occur. I just wanted to know that from the medical literature and then it would be of course enormously helpful if prepared medical literature is available on such a [web]side, that would be in my opinion a kind of value add to such a [web]side. I mean PREPARED. So to speak, there is this study and now once, it is explained for nonprofessionals in an understandable manner, what is the study about. Maybe summarized on one page, like such an abstract. And (...) below it, however, also the primary source. Because not everyone has the possibility to access the university library, so to speak."* (Patient #3) - *„Yes, exactly, because this is also an ongoing process. So, now what do I know about these immune adjuvant immunotherapies, you learn constantly more, also in the medical world. Yes, that’s it, there is research [going on], there are new developments, there are new findings, there are perhaps sometime new drugs or new forms of therapy [available], etc.. And (..) yes, to be able to participate in this knowledge. Let’s say, a little bit like Drosten [=Christian Drosten, German virologist] did with Corona. I think his greatest merit is that he translates the state of science (..) for the general public in such a way that you can understand it with a little bit of good will. But he keeps up with the current developments. He also sometimes says, we don't know yet, we're doing research, a new study has just been published, but I evaluate it this way and that way, maybe it's a bit too small or it's solid. You can gain this knowledge from that and this constantly, so to speak, taking the affected people along on this, well, scientific journey, so to speak, [but] this [kind of] translation performance, I've hardly ever found that in the field of skin cancer. There is the scientific world with all its studies and a lot of Latin and Greek and then there is, so to speak, what is sweated out at the bottom and what, let’s say, “Krethi and Plethi” [=German equivalent to “every Tom, Dick and Harry”] may bear to hear. And in between (...) there is no, no firmly established transmission belt. And I could well imagine this, well, as an additional value of such a page. It means that it is not built up once and then activated and that's it for 10 years, but that you would also constantly work on it.”* (Patient #3) - *„[Establish a category] “current developments” or “current findings”, whatsoever, and you could incorporate something like that very well, exactly what is discussed at congresses, what is now being tried and tested somewhere in the world, I don't know. But you would have to edit it [for nonprofessionals]“* (Patient #3) |
|  | **No contact address was available, which limits the website’s professionalism and trustworthiness**   - „*So, as I said, I just feel comfortable when I'm on a website and when I click there and then I know, ok, there's a head office, it's in Erlangen or Dresden or somewhere, then I know, ok, this thing is reliable.*“(Patient #5) |
