# Supplementary material for: One Website to Gather them All: Usability Testing of the New German SKin Cancer INFOrmation (SKINFO) Website—A Mixed-methods Approach
Source: J Cancer Educ. 2022 Dec 30;38(4):1264–70. doi: 10.1007/s13187-022-02258-5 (PMC10366310; doi:10.1007/s13187-022-02258-5)
Supplement: Supplementary file 4 — (DOCX 19 kb) [file 13187_2022_2258_MOESM4_ESM.docx]

**Supplementary Table 3:** Mean values per item of the user experience questionnaire.

| **Item** | **Mean** | **Std. Dev.** | **Left** | **Right** | **Scale** |
| --- | --- | --- | --- | --- | --- |
| 1 | 2.1 | 1.5 | annoying | enjoyable | attractiveness |
| 2 | 2.1 | 0.9 | not understandable | understandable | perspicuity |
| 3 | 1.7 | 1.1 | creative | dull | novelty |
| 4 | 1.6 | 1.6 | easy to learn | difficult to learn | perspicuity |
| 5 | 2.7 | 0.7 | valuable | inferior | stimulation |
| 6 | 1.9 | 1.1 | boring | exciting | stimulation |
| 7 | 2.3 | 0.8 | not interesting | interesting | stimulation |
| 8 | 1.4 | 1.2 | unpredictable | predictable | dependability |
| 9 | 2.0 | 1.2 | fast | slow | efficiency |
| 10 | 0.6 | 1.4 | inventive | conventional | novelty |
| 11 | 2.6 | 0.5 | obstructive | supportive | dependability |
| 12 | 2.2 | 1.5 | good | bad | attractiveness |
| 13 | 1.1 | 1.8 | complicated | easy | perspicuity |
| 14 | 1.8 | 1.1 | unlikable | pleasing | attractiveness |
| 15 | 1.1 | 1.8 | usual | leading edge | novelty |
| 16 | 1.9 | 1.3 | unpleasant | pleasant | attractiveness |
| 17 | 2.2 | 1.3 | secure | not secure | dependability |
| 18 | 1.8 | 1.1 | motivating | demotivating | stimulation |
| 19 | 1.9 | 1.0 | meets expectations | does not meet expectations | dependability |
| 20 | 1.8 | 1.0 | inefficient | efficient | efficiency |
| 21 | 1.7 | 1.5 | clear | confusing | perspicuity |
| 22 | 1.4 | 1.8 | impractical | practical | efficiency |
| 23 | 2.4 | 1.3 | organized | cluttered | efficiency |
| 24 | 2.1 | 1.0 | attractive | unattractive | attractiveness |
| 25 | 1.9 | 0.9 | friendly | unfriendly | attractiveness |
| 26 | 0.9 | 1.2 | conservative | innovative | novelty |
